# Supplementary material for: Modeling glioblastoma heterogeneity as a dynamic network of cell states
Source: Mol Syst Biol. 2021 Sep 16;17(9):e10105. doi: 10.15252/msb.202010105 (PMC8444284; doi:10.15252/msb.202010105)
Supplement: Supplementary file 5 — Source Data for Figure 3 [file MSB-17-e10105-s001.zip › Figure3A_sourcedata/GSEA_3065/nosigdb_state4.GseaPreranked.1622718430994/FELDMAN_2020_S-G2.html]

Details for gene set FELDMAN\_2020\_S-G2[GSEA]

|  || Dataset | state4 |
| Phenotype | NoPhenotypeAvailable |
| Upregulated in class | na\_neg |
| GeneSet | FELDMAN\_2020\_S-G2 |
| Enrichment Score (ES) | -0.70921797 |
| Normalized Enrichment Score (NES) | -2.8650546 |
| Nominal p-value | 0.0 |
| FDR q-value | 0.0 |
| FWER p-Value | 0.0 |
Table: GSEA Results Summary

  

Fig 1: Enrichment plot: FELDMAN\_2020\_S-G2      
 Profile of the Running ES Score & Positions of GeneSet Members on the Rank Ordered List

  

| PROBE | GENE SYMBOL | GENE\_TITLE | RANK IN GENE LIST | RANK METRIC SCORE | RUNNING ES | CORE ENRICHMENT || 1 | ID3 |  |  | 2 | 0.730 | 0.0086 | No |
| 2 | SAT1 |  |  | 4 | 0.669 | 0.0167 | No |
| 3 | FABP7 |  |  | 5 | 0.656 | 0.0248 | No |
| 4 | S100B |  |  | 6 | 0.630 | 0.0325 | No |
| 5 | BEX1 |  |  | 11 | 0.473 | 0.0377 | No |
| 6 | EEF1A1 |  |  | 12 | 0.471 | 0.0435 | No |
| 7 | FTL |  |  | 13 | 0.466 | 0.0492 | No |
| 8 | TUBA1A |  |  | 15 | 0.460 | 0.0547 | No |
| 9 | SOX4 |  |  | 18 | 0.402 | 0.0593 | No |
| 10 | COMMD6 |  |  | 36 | 0.334 | 0.0605 | No |
| 11 | C4orf3 |  |  | 43 | 0.308 | 0.0633 | No |
| 12 | ATP6V1G1 |  |  | 47 | 0.303 | 0.0665 | No |
| 13 | SOX9 |  |  | 48 | 0.299 | 0.0702 | No |
| 14 | SESN3 |  |  | 68 | 0.278 | 0.0704 | No |
| 15 | RPL34 |  |  | 69 | 0.278 | 0.0739 | No |
| 16 | PFDN5 |  |  | 78 | 0.265 | 0.0758 | No |
| 17 | CLU |  |  | 95 | 0.252 | 0.0762 | No |
| 18 | TTYH1 |  |  | 100 | 0.249 | 0.0786 | No |
| 19 | RPS27L |  |  | 149 | 0.223 | 0.0732 | No |
| 20 | PCSK1N |  |  | 172 | 0.212 | 0.0721 | No |
| 21 | SDCBP |  |  | 212 | 0.201 | 0.0680 | No |
| 22 | BEX4 |  |  | 220 | 0.194 | 0.0693 | No |
| 23 | LYPD1 |  |  | 264 | 0.183 | 0.0643 | No |
| 24 | MLLT11 |  |  | 267 | 0.182 | 0.0662 | No |
| 25 | CDKN1A |  |  | 319 | 0.171 | 0.0597 | No |
| 26 | PLP1 |  |  | 330 | 0.169 | 0.0601 | No |
| 27 | GPM6B |  |  | 346 | 0.166 | 0.0596 | No |
| 28 | ST13 |  |  | 372 | 0.159 | 0.0574 | No |
| 29 | WDR83OS |  |  | 415 | 0.149 | 0.0521 | No |
| 30 | PRDX1 |  |  | 416 | 0.149 | 0.0540 | No |
| 31 | PHLDA1 |  |  | 464 | 0.142 | 0.0478 | No |
| 32 | DDIT4 |  |  | 493 | 0.138 | 0.0448 | No |
| 33 | SPARC |  |  | 539 | 0.132 | 0.0389 | No |
| 34 | IFITM3 |  |  | 546 | 0.132 | 0.0395 | No |
| 35 | PSAP |  |  | 596 | 0.126 | 0.0328 | No |
| 36 | PLD3 |  |  | 666 | 0.118 | 0.0226 | No |
| 37 | EEF2 |  |  | 729 | 0.110 | 0.0136 | No |
| 38 | YPEL5 |  |  | 783 | 0.105 | 0.0059 | No |
| 39 | RNF19A |  |  | 793 | 0.104 | 0.0057 | No |
| 40 | IGFBP5 |  |  | 901 | 0.096 | -0.0111 | No |
| 41 | EPS8L1 |  |  | 913 | 0.094 | -0.0118 | No |
| 42 | MAGED2 |  |  | 946 | 0.092 | -0.0160 | No |
| 43 | ZFAND6 |  |  | 978 | 0.090 | -0.0202 | No |
| 44 | ENC1 |  |  | 1024 | 0.086 | -0.0267 | No |
| 45 | HES4 |  |  | 1197 | 0.076 | -0.0546 | No |
| 46 | RAB13 |  |  | 1270 | 0.072 | -0.0659 | No |
| 47 | SCD5 |  |  | 1290 | 0.071 | -0.0682 | No |
| 48 | EIF4A2 |  |  | 1311 | 0.070 | -0.0707 | No |
| 49 | PRCP |  |  | 1675 | 0.056 | -0.1310 | No |
| 50 | TM7SF2 |  |  | 1689 | 0.056 | -0.1325 | No |
| 51 | NDFIP1 |  |  | 2664 | 0.031 | -0.2960 | No |
| 52 | BAMBI |  |  | 3437 | 0.014 | -0.4256 | No |
| 53 | SNRNP25 |  |  | 3634 | 0.008 | -0.4585 | No |
| 54 | TUBA1B |  |  | 3658 | 0.007 | -0.4623 | No |
| 55 | CALM2 |  |  | 3844 | -0.001 | -0.4934 | No |
| 56 | RABAC1 |  |  | 3855 | -0.002 | -0.4950 | No |
| 57 | KLF6 |  |  | 3920 | -0.005 | -0.5057 | No |
| 58 | CHCHD2 |  |  | 3928 | -0.006 | -0.5068 | No |
| 59 | BTG1 |  |  | 4064 | -0.013 | -0.5294 | No |
| 60 | RBM17 |  |  | 4322 | -0.031 | -0.5722 | No |
| 61 | PRELID1 |  |  | 4327 | -0.031 | -0.5725 | No |
| 62 | ARL6IP5 |  |  | 4364 | -0.033 | -0.5782 | No |
| 63 | TFDP1 |  |  | 4389 | -0.035 | -0.5818 | No |
| 64 | MIAT |  |  | 4438 | -0.039 | -0.5894 | No |
| 65 | RRS1 |  |  | 4482 | -0.043 | -0.5961 | No |
| 66 | MRPL18 |  |  | 4484 | -0.043 | -0.5957 | No |
| 67 | CNPY2 |  |  | 4540 | -0.047 | -0.6044 | No |
| 68 | PFDN2 |  |  | 4562 | -0.049 | -0.6073 | No |
| 69 | NUDT15 |  |  | 4571 | -0.049 | -0.6081 | No |
| 70 | YWHAH |  |  | 4595 | -0.051 | -0.6113 | No |
| 71 | RMI2 |  |  | 4623 | -0.053 | -0.6152 | No |
| 72 | EIF1AX |  |  | 4700 | -0.060 | -0.6272 | No |
| 73 | CMTM7 |  |  | 4735 | -0.063 | -0.6322 | No |
| 74 | RAD23A |  |  | 4743 | -0.063 | -0.6326 | No |
| 75 | HSPE1 |  |  | 4744 | -0.063 | -0.6318 | No |
| 76 | COPS3 |  |  | 4758 | -0.065 | -0.6332 | No |
| 77 | HNRNPF |  |  | 4783 | -0.066 | -0.6364 | No |
| 78 | CDH2 |  |  | 4839 | -0.071 | -0.6448 | No |
| 79 | FOSL1 |  |  | 4845 | -0.072 | -0.6448 | No |
| 80 | PIN1 |  |  | 4886 | -0.074 | -0.6506 | No |
| 81 | CYCS |  |  | 4894 | -0.075 | -0.6508 | No |
| 82 | POLR3K |  |  | 4904 | -0.075 | -0.6514 | No |
| 83 | CDKN2D |  |  | 4933 | -0.077 | -0.6552 | No |
| 84 | MRPL17 |  |  | 4937 | -0.078 | -0.6547 | No |
| 85 | SCRG1 |  |  | 4970 | -0.080 | -0.6591 | No |
| 86 | ACYP1 |  |  | 4975 | -0.081 | -0.6588 | No |
| 87 | EIF5 |  |  | 4991 | -0.082 | -0.6603 | No |
| 88 | SET |  |  | 4997 | -0.082 | -0.6601 | No |
| 89 | HSPA8 |  |  | 5041 | -0.086 | -0.6663 | No |
| 90 | ITM2B |  |  | 5050 | -0.087 | -0.6666 | No |
| 91 | DONSON |  |  | 5051 | -0.087 | -0.6655 | No |
| 92 | PARP1 |  |  | 5065 | -0.088 | -0.6666 | No |
| 93 | DCK |  |  | 5068 | -0.088 | -0.6659 | No |
| 94 | GGCT |  |  | 5073 | -0.089 | -0.6655 | No |
| 95 | TUBB6 |  |  | 5076 | -0.089 | -0.6647 | No |
| 96 | CARHSP1 |  |  | 5107 | -0.092 | -0.6686 | No |
| 97 | PARP2 |  |  | 5119 | -0.093 | -0.6693 | No |
| 98 | SKA3 |  |  | 5136 | -0.095 | -0.6708 | No |
| 99 | SNRNP40 |  |  | 5174 | -0.099 | -0.6758 | No |
| 100 | SUPT16H |  |  | 5179 | -0.099 | -0.6753 | No |
| 101 | BRIX1 |  |  | 5203 | -0.102 | -0.6779 | No |
| 102 | HMGN1 |  |  | 5223 | -0.103 | -0.6798 | No |
| 103 | RNASEH2A |  |  | 5241 | -0.105 | -0.6814 | No |
| 104 | CMSS1 |  |  | 5247 | -0.105 | -0.6809 | No |
| 105 | POP7 |  |  | 5259 | -0.107 | -0.6815 | No |
| 106 | TMEM59 |  |  | 5275 | -0.108 | -0.6827 | No |
| 107 | UBE2I |  |  | 5293 | -0.109 | -0.6842 | No |
| 108 | RHEB |  |  | 5346 | -0.113 | -0.6916 | No |
| 109 | PDCD5 |  |  | 5366 | -0.115 | -0.6933 | No |
| 110 | H2AFV |  |  | 5381 | -0.117 | -0.6943 | No |
| 111 | CKLF |  |  | 5394 | -0.118 | -0.6948 | No |
| 112 | RFC5 |  |  | 5434 | -0.122 | -0.6999 | No |
| 113 | LRR1 |  |  | 5459 | -0.126 | -0.7024 | No |
| 114 | TUBB |  |  | 5482 | -0.129 | -0.7045 | No |
| 115 | WDR34 |  |  | 5484 | -0.130 | -0.7030 | No |
| 116 | KPNB1 |  |  | 5514 | -0.133 | -0.7063 | No |
| 117 | PSMG1 |  |  | 5518 | -0.133 | -0.7052 | No |
| 118 | SNRPG |  |  | 5530 | -0.134 | -0.7053 | No |
| 119 | HPRT1 |  |  | 5554 | -0.136 | -0.7075 | Yes |
| 120 | SPDL1 |  |  | 5560 | -0.137 | -0.7067 | Yes |
| 121 | GPM6A |  |  | 5563 | -0.137 | -0.7053 | Yes |
| 122 | MAP3K7CL |  |  | 5565 | -0.138 | -0.7038 | Yes |
| 123 | BUB3 |  |  | 5583 | -0.141 | -0.7049 | Yes |
| 124 | CENPW |  |  | 5585 | -0.142 | -0.7034 | Yes |
| 125 | DCTPP1 |  |  | 5588 | -0.143 | -0.7019 | Yes |
| 126 | CHCHD3 |  |  | 5589 | -0.143 | -0.7002 | Yes |
| 127 | PSIP1 |  |  | 5590 | -0.143 | -0.6984 | Yes |
| 128 | SLC25A5 |  |  | 5628 | -0.148 | -0.7028 | Yes |
| 129 | TEX30 |  |  | 5629 | -0.148 | -0.7010 | Yes |
| 130 | HIRIP3 |  |  | 5638 | -0.151 | -0.7005 | Yes |
| 131 | KIFC1 |  |  | 5645 | -0.152 | -0.6996 | Yes |
| 132 | CCDC34 |  |  | 5647 | -0.152 | -0.6979 | Yes |
| 133 | TRA2B |  |  | 5652 | -0.152 | -0.6967 | Yes |
| 134 | SNRPE |  |  | 5653 | -0.152 | -0.6949 | Yes |
| 135 | HIST2H2AC |  |  | 5670 | -0.155 | -0.6956 | Yes |
| 136 | LSM4 |  |  | 5673 | -0.155 | -0.6941 | Yes |
| 137 | RNPS1 |  |  | 5685 | -0.157 | -0.6940 | Yes |
| 138 | DSN1 |  |  | 5687 | -0.158 | -0.6922 | Yes |
| 139 | TUBG1 |  |  | 5690 | -0.158 | -0.6906 | Yes |
| 140 | HNRNPD |  |  | 5705 | -0.161 | -0.6910 | Yes |
| 141 | HSPB11 |  |  | 5707 | -0.161 | -0.6892 | Yes |
| 142 | CACYBP |  |  | 5713 | -0.162 | -0.6880 | Yes |
| 143 | MAGOHB |  |  | 5718 | -0.164 | -0.6867 | Yes |
| 144 | IER2 |  |  | 5719 | -0.164 | -0.6846 | Yes |
| 145 | EIF4EBP1 |  |  | 5747 | -0.171 | -0.6871 | Yes |
| 146 | EXOSC8 |  |  | 5751 | -0.173 | -0.6855 | Yes |
| 147 | CISD2 |  |  | 5764 | -0.176 | -0.6853 | Yes |
| 148 | FHL2 |  |  | 5776 | -0.179 | -0.6850 | Yes |
| 149 | SNRPA1 |  |  | 5780 | -0.179 | -0.6833 | Yes |
| 150 | MALAT1 |  |  | 5783 | -0.179 | -0.6814 | Yes |
| 151 | SIVA1 |  |  | 5791 | -0.181 | -0.6804 | Yes |
| 152 | TRIP13 |  |  | 5792 | -0.181 | -0.6781 | Yes |
| 153 | TCF19 |  |  | 5796 | -0.182 | -0.6764 | Yes |
| 154 | HAT1 |  |  | 5803 | -0.184 | -0.6751 | Yes |
| 155 | ZWILCH |  |  | 5805 | -0.184 | -0.6731 | Yes |
| 156 | SAC3D1 |  |  | 5810 | -0.186 | -0.6714 | Yes |
| 157 | PTPRZ1 |  |  | 5811 | -0.186 | -0.6691 | Yes |
| 158 | MIS18A |  |  | 5816 | -0.187 | -0.6675 | Yes |
| 159 | LAPTM4A |  |  | 5819 | -0.187 | -0.6656 | Yes |
| 160 | HOXB2 |  |  | 5825 | -0.189 | -0.6641 | Yes |
| 161 | IL11 |  |  | 5830 | -0.190 | -0.6624 | Yes |
| 162 | PSMC3 |  |  | 5831 | -0.190 | -0.6601 | Yes |
| 163 | CMC2 |  |  | 5834 | -0.191 | -0.6581 | Yes |
| 164 | PRC1 |  |  | 5835 | -0.192 | -0.6557 | Yes |
| 165 | PA2G4 |  |  | 5841 | -0.193 | -0.6542 | Yes |
| 166 | PTMA |  |  | 5852 | -0.195 | -0.6535 | Yes |
| 167 | RBBP8 |  |  | 5854 | -0.195 | -0.6512 | Yes |
| 168 | PAICS |  |  | 5858 | -0.196 | -0.6493 | Yes |
| 169 | CDCA4 |  |  | 5861 | -0.197 | -0.6472 | Yes |
| 170 | HNRNPAB |  |  | 5862 | -0.197 | -0.6448 | Yes |
| 171 | HIST1H1E |  |  | 5865 | -0.198 | -0.6427 | Yes |
| 172 | PMAIP1 |  |  | 5871 | -0.199 | -0.6411 | Yes |
| 173 | CKAP2 |  |  | 5879 | -0.201 | -0.6398 | Yes |
| 174 | SAE1 |  |  | 5888 | -0.203 | -0.6386 | Yes |
| 175 | ANP32B |  |  | 5890 | -0.204 | -0.6363 | Yes |
| 176 | DIAPH3 |  |  | 5893 | -0.205 | -0.6341 | Yes |
| 177 | C19orf48 |  |  | 5894 | -0.205 | -0.6316 | Yes |
| 178 | BTG3 |  |  | 5895 | -0.206 | -0.6291 | Yes |
| 179 | C12orf75 |  |  | 5896 | -0.206 | -0.6265 | Yes |
| 180 | SNRPB |  |  | 5908 | -0.209 | -0.6258 | Yes |
| 181 | BRCA2 |  |  | 5910 | -0.210 | -0.6234 | Yes |
| 182 | SNRPD1 |  |  | 5911 | -0.210 | -0.6208 | Yes |
| 183 | PTGES3 |  |  | 5912 | -0.210 | -0.6183 | Yes |
| 184 | RANBP1 |  |  | 5939 | -0.218 | -0.6199 | Yes |
| 185 | SMS |  |  | 5940 | -0.218 | -0.6173 | Yes |
| 186 | TOMM40 |  |  | 5950 | -0.221 | -0.6161 | Yes |
| 187 | PSMC3IP |  |  | 5951 | -0.222 | -0.6133 | Yes |
| 188 | CENPA |  |  | 5960 | -0.226 | -0.6119 | Yes |
| 189 | TMEM237 |  |  | 5962 | -0.226 | -0.6093 | Yes |
| 190 | PGP |  |  | 5966 | -0.227 | -0.6070 | Yes |
| 191 | HSPD1 |  |  | 5967 | -0.227 | -0.6042 | Yes |
| 192 | SRSF3 |  |  | 5969 | -0.227 | -0.6016 | Yes |
| 193 | NCL |  |  | 5974 | -0.228 | -0.5994 | Yes |
| 194 | RTKN2 |  |  | 5977 | -0.229 | -0.5970 | Yes |
| 195 | TMEM106C |  |  | 5979 | -0.230 | -0.5943 | Yes |
| 196 | MND1 |  |  | 5982 | -0.232 | -0.5918 | Yes |
| 197 | CENPV |  |  | 5984 | -0.232 | -0.5891 | Yes |
| 198 | USP1 |  |  | 5986 | -0.233 | -0.5864 | Yes |
| 199 | AKAP12 |  |  | 5988 | -0.234 | -0.5837 | Yes |
| 200 | MZT1 |  |  | 5989 | -0.235 | -0.5808 | Yes |
| 201 | RAN |  |  | 5990 | -0.235 | -0.5779 | Yes |
| 202 | STMN1 |  |  | 5993 | -0.236 | -0.5753 | Yes |
| 203 | RPA3 |  |  | 6014 | -0.244 | -0.5757 | Yes |
| 204 | UPP1 |  |  | 6016 | -0.245 | -0.5729 | Yes |
| 205 | SMC1A |  |  | 6017 | -0.245 | -0.5699 | Yes |
| 206 | CTNNAL1 |  |  | 6027 | -0.249 | -0.5683 | Yes |
| 207 | HNRNPDL |  |  | 6028 | -0.251 | -0.5652 | Yes |
| 208 | DEPDC1 |  |  | 6030 | -0.251 | -0.5623 | Yes |
| 209 | CEP78 |  |  | 6031 | -0.253 | -0.5592 | Yes |
| 210 | VRK1 |  |  | 6034 | -0.256 | -0.5564 | Yes |
| 211 | FEN1 |  |  | 6035 | -0.256 | -0.5532 | Yes |
| 212 | DNAJC9 |  |  | 6037 | -0.260 | -0.5502 | Yes |
| 213 | SMC3 |  |  | 6041 | -0.265 | -0.5474 | Yes |
| 214 | MXD3 |  |  | 6042 | -0.265 | -0.5442 | Yes |
| 215 | NETO2 |  |  | 6043 | -0.266 | -0.5409 | Yes |
| 216 | HMGB1 |  |  | 6047 | -0.267 | -0.5381 | Yes |
| 217 | HJURP |  |  | 6055 | -0.271 | -0.5360 | Yes |
| 218 | HSP90AA1 |  |  | 6062 | -0.275 | -0.5336 | Yes |
| 219 | CDC45 |  |  | 6063 | -0.276 | -0.5302 | Yes |
| 220 | ARHGAP11A |  |  | 6067 | -0.277 | -0.5273 | Yes |
| 221 | SHCBP1 |  |  | 6071 | -0.279 | -0.5244 | Yes |
| 222 | SPC25 |  |  | 6072 | -0.279 | -0.5209 | Yes |
| 223 | CEP55 |  |  | 6076 | -0.281 | -0.5180 | Yes |
| 224 | HIST1H1C |  |  | 6077 | -0.284 | -0.5145 | Yes |
| 225 | PLAT |  |  | 6079 | -0.285 | -0.5112 | Yes |
| 226 | GMNN |  |  | 6080 | -0.285 | -0.5076 | Yes |
| 227 | PLP2 |  |  | 6082 | -0.286 | -0.5043 | Yes |
| 228 | FBXO5 |  |  | 6084 | -0.287 | -0.5009 | Yes |
| 229 | SYNE2 |  |  | 6086 | -0.287 | -0.4976 | Yes |
| 230 | KIF22 |  |  | 6089 | -0.290 | -0.4943 | Yes |
| 231 | CENPN |  |  | 6090 | -0.291 | -0.4908 | Yes |
| 232 | DEK |  |  | 6092 | -0.292 | -0.4873 | Yes |
| 233 | NUF2 |  |  | 6093 | -0.293 | -0.4837 | Yes |
| 234 | RFC2 |  |  | 6095 | -0.294 | -0.4803 | Yes |
| 235 | CCNA2 |  |  | 6100 | -0.297 | -0.4773 | Yes |
| 236 | DCBLD2 |  |  | 6104 | -0.300 | -0.4741 | Yes |
| 237 | DUT |  |  | 6107 | -0.303 | -0.4707 | Yes |
| 238 | CKAP2L |  |  | 6108 | -0.303 | -0.4670 | Yes |
| 239 | MCM3 |  |  | 6113 | -0.307 | -0.4639 | Yes |
| 240 | MELK |  |  | 6117 | -0.312 | -0.4606 | Yes |
| 241 | MIS18BP1 |  |  | 6122 | -0.316 | -0.4574 | Yes |
| 242 | ASF1B |  |  | 6127 | -0.320 | -0.4541 | Yes |
| 243 | SRSF2 |  |  | 6128 | -0.321 | -0.4502 | Yes |
| 244 | HMGA1 |  |  | 6132 | -0.324 | -0.4467 | Yes |
| 245 | LMNB1 |  |  | 6134 | -0.325 | -0.4429 | Yes |
| 246 | CDCA2 |  |  | 6135 | -0.326 | -0.4388 | Yes |
| 247 | CENPH |  |  | 6136 | -0.327 | -0.4348 | Yes |
| 248 | TACC3 |  |  | 6138 | -0.328 | -0.4310 | Yes |
| 249 | NDC80 |  |  | 6142 | -0.330 | -0.4274 | Yes |
| 250 | AURKB |  |  | 6143 | -0.332 | -0.4233 | Yes |
| 251 | TK1 |  |  | 6144 | -0.334 | -0.4192 | Yes |
| 252 | NUDT1 |  |  | 6146 | -0.338 | -0.4152 | Yes |
| 253 | RRM1 |  |  | 6147 | -0.338 | -0.4111 | Yes |
| 254 | PRKDC |  |  | 6149 | -0.341 | -0.4070 | Yes |
| 255 | SRSF7 |  |  | 6150 | -0.343 | -0.4028 | Yes |
| 256 | ATAD5 |  |  | 6161 | -0.350 | -0.4002 | Yes |
| 257 | KIF23 |  |  | 6162 | -0.351 | -0.3959 | Yes |
| 258 | HMGN2 |  |  | 6163 | -0.351 | -0.3916 | Yes |
| 259 | KIF4A |  |  | 6164 | -0.355 | -0.3872 | Yes |
| 260 | CDCA3 |  |  | 6165 | -0.355 | -0.3828 | Yes |
| 261 | RAD51AP1 |  |  | 6167 | -0.357 | -0.3786 | Yes |
| 262 | CENPK |  |  | 6169 | -0.363 | -0.3743 | Yes |
| 263 | KIF20B |  |  | 6172 | -0.373 | -0.3701 | Yes |
| 264 | PCNA |  |  | 6173 | -0.374 | -0.3655 | Yes |
| 265 | DTYMK |  |  | 6177 | -0.381 | -0.3613 | Yes |
| 266 | CENPM |  |  | 6179 | -0.386 | -0.3567 | Yes |
| 267 | DBF4 |  |  | 6180 | -0.387 | -0.3519 | Yes |
| 268 | DNMT1 |  |  | 6182 | -0.396 | -0.3472 | Yes |
| 269 | IGFBP3 |  |  | 6184 | -0.397 | -0.3425 | Yes |
| 270 | RFC4 |  |  | 6185 | -0.399 | -0.3376 | Yes |
| 271 | NASP |  |  | 6187 | -0.403 | -0.3328 | Yes |
| 272 | TMEM158 |  |  | 6189 | -0.407 | -0.3280 | Yes |
| 273 | MYBL2 |  |  | 6190 | -0.408 | -0.3230 | Yes |
| 274 | GINS2 |  |  | 6194 | -0.420 | -0.3183 | Yes |
| 275 | ZWINT |  |  | 6195 | -0.425 | -0.3131 | Yes |
| 276 | TUBB4B |  |  | 6196 | -0.431 | -0.3078 | Yes |
| 277 | RRM2 |  |  | 6199 | -0.438 | -0.3027 | Yes |
| 278 | DHFR |  |  | 6200 | -0.441 | -0.2973 | Yes |
| 279 | ESCO2 |  |  | 6206 | -0.463 | -0.2925 | Yes |
| 280 | PBK |  |  | 6207 | -0.465 | -0.2867 | Yes |
| 281 | BIRC5 |  |  | 6214 | -0.484 | -0.2818 | Yes |
| 282 | ANLN |  |  | 6215 | -0.484 | -0.2758 | Yes |
| 283 | GTSE1 |  |  | 6217 | -0.490 | -0.2700 | Yes |
| 284 | CLSPN |  |  | 6218 | -0.490 | -0.2640 | Yes |
| 285 | SMC4 |  |  | 6219 | -0.490 | -0.2579 | Yes |
| 286 | HELLS |  |  | 6221 | -0.501 | -0.2519 | Yes |
| 287 | TYMS |  |  | 6222 | -0.501 | -0.2458 | Yes |
| 288 | NUSAP1 |  |  | 6223 | -0.507 | -0.2395 | Yes |
| 289 | CENPE |  |  | 6226 | -0.511 | -0.2336 | Yes |
| 290 | NCAPG |  |  | 6227 | -0.513 | -0.2273 | Yes |
| 291 | SMC2 |  |  | 6229 | -0.518 | -0.2211 | Yes |
| 292 | GGH |  |  | 6232 | -0.522 | -0.2150 | Yes |
| 293 | TMPO |  |  | 6233 | -0.522 | -0.2086 | Yes |
| 294 | CKS1B |  |  | 6234 | -0.523 | -0.2021 | Yes |
| 295 | KPNA2 |  |  | 6236 | -0.543 | -0.1956 | Yes |
| 296 | H2AFX |  |  | 6237 | -0.576 | -0.1885 | Yes |
| 297 | ORC6 |  |  | 6238 | -0.585 | -0.1813 | Yes |
| 298 | CKS2 |  |  | 6239 | -0.585 | -0.1741 | Yes |
| 299 | UBE2T |  |  | 6240 | -0.593 | -0.1668 | Yes |
| 300 | HIST1H1D |  |  | 6241 | -0.601 | -0.1594 | Yes |
| 301 | ATAD2 |  |  | 6243 | -0.607 | -0.1521 | Yes |
| 302 | UBE2S |  |  | 6244 | -0.617 | -0.1446 | Yes |
| 303 | PTTG1 |  |  | 6245 | -0.622 | -0.1369 | Yes |
| 304 | CDK1 |  |  | 6246 | -0.627 | -0.1292 | Yes |
| 305 | MAD2L1 |  |  | 6247 | -0.628 | -0.1215 | Yes |
| 306 | CENPU |  |  | 6248 | -0.633 | -0.1137 | Yes |
| 307 | HIST1H1B |  |  | 6249 | -0.646 | -0.1058 | Yes |
| 308 | H2AFZ |  |  | 6250 | -0.651 | -0.0977 | Yes |
| 309 | TNFRSF12A |  |  | 6251 | -0.658 | -0.0896 | Yes |
| 310 | MCM7 |  |  | 6252 | -0.665 | -0.0815 | Yes |
| 311 | TPX2 |  |  | 6253 | -0.667 | -0.0733 | Yes |
| 312 | ASPM |  |  | 6254 | -0.680 | -0.0649 | Yes |
| 313 | UBE2C |  |  | 6255 | -0.750 | -0.0557 | Yes |
| 314 | HMGB2 |  |  | 6258 | -0.766 | -0.0466 | Yes |
| 315 | TOP2A |  |  | 6260 | -0.814 | -0.0367 | Yes |
| 316 | MKI67 |  |  | 6261 | -0.882 | -0.0259 | Yes |
| 317 | HIST1H4C |  |  | 6262 | -1.038 | -0.0131 | Yes |
| 318 | CENPF |  |  | 6263 | -1.068 | 0.0000 | Yes |
Table: GSEA details [plain text format]

  

Fig 2: FELDMAN\_2020\_S-G2: Random ES distribution      
 Gene set null distribution of ES for **FELDMAN\_2020\_S-G2**
